# Profil clinique, biologique et radiologique des patients Algériens hospitalisés pour COVID-19: données préliminaires

**DOI:** 10.11604/pamj.supp.2020.35.2.23807

**Published:** 2020-06-15

**Authors:** Abdelbassat Ketfi, Omar Chabati, Samia Chemali, Mohamed Mahjoub, Merzak Gharnaout, Rama Touahri, Kamel Djenouhat, Fayçal Selatni, Helmi Ben Saad

**Affiliations:** 1 Service de Pneumologie, de Phtisiologie et d´Allergologie(SPPA), Hôpital de Rouiba, Université d´Alger 1, Faculté de Médecine d´Alger, Alger, Algérie; 2 Service de Médecine Interne, Hôpital de Rouiba, Université d´Alger 1, Faculté de Médecine d´Alger, Alger, Algérie; 3 Université de Sousse, Hôpital Farhat HACHED, Service d´hygiène hospitalière. Sousse, Tunisie; 4 Service de biologie médicale, Hôpital de Rouiba, Université d´Alger 1, Faculté de Médecine d´Alger, Alger, Algérie; 5 Centre d´imagerie médicale (Cimagerie), Rouiba, Alger, Algérie; 6 Université de Sousse, Hôpital Farhat HACHED, Service de Physiologie et Explorations Fonctionnelles, Sousse, Tunisie; 7 Université de Sousse, Faculté de Médecine de Sousse, Laboratoire de Physiologie, Tunisie; 8 Laboratoire de recherche “Insuffisance Cardiaque, LR12SP09”, EPS Farhat HACHED, Sousse, Tunisie

**Keywords:** Coronavirus, pneumonie, COVID-19, profil des patients, Afrique du Nord

## Abstract

**Introduction:**

Aucune étude antérieure n’a élaboré le profil des patients Algériens hospitalisés pour COVID-19. L’objectif de cette étude était de déterminer le profil clinique, biologique et tomodensitométrique des patients Algériens hospitalisés pour COVID-19.

**Méthodes:**

Une étude prospective était menée auprès des patients hospitalisés pour COVID-19 (période: 19 mars-30 avril 2020). Les données cliniques, biologiques et radiologiques, le type de traitement reçu et la durée de l’hospitalisation étaient notés.

**Résultats:**

Le profil clinique des 86 patients atteints de COVID-19 était un homme non-fumeur, âgé de 53 ans, qui était dans 42% des cas en contact avec un cas suspect/confirmé de COVID-19 et ayant une comorbidité dans 70% des cas (hypertension artérielle, diabète sucré, pathologie respiratoire chronique et allergie, cardiopathie). Les plaintes cliniques étaient dominées par la triade «asthénie-fièvre-toux» dans plus de 70% des cas. Les anomalies biologiques les plus fréquentes étaient: syndrome inflammatoire biologique (90,1%), basocytémie (70,8%), lymphopénie (53,3%), augmentation de la lactico-deshydrogénase (52,2%), anémie (38,7%), augmentation de la phosphokinase (28,8%) et cytolyse hépatique (27,6%). Les signes tomodensitométriques les plus fréquents étaient: verre dépoli (91,8%), condensations alvéolaires (61,2%), verre dépoli en plage (60,0%), et verre dépoli nodulaire (55,3%). Un traitement à base de «chloroquine, azithromycine, zinc, vitamine C, enoxaparine, double antibiothérapie et ± corticoïdes» était prescrit chez 34,9% des patients. La moyenne de la durée d’hospitalisation était de 7±3 jours.

**Conclusion:**

La connaissance des profils des formes modérées et sévères du COVID-19 contribuerait à faire progresser les stratégies de contrôle de l’infection en Algérie.

## Introduction

En décembre 2019, une épidémie de pneumonie due au nouveau coronavirus 2019, le SARS-CoV-2 (severe acute respiratory syndrome coronavirus 2) a éclaté à Wuhan, Hubei, Chine [1]. Ce bétacoronavirus provoque une pathologie respiratoire parfois sévère, nommée COVID-19 par l´organisation mondiale de la santé (OMS). Le 12 mars 2020, l’OMS a déclaré le COVID-19 comme une pandémie [1-3]. En effet, après l´Asie, l´Europe, les États-Unis et l´Iran sont les régions du monde les plus touchées [3]. A la date du 28 mai 2020, le nombre de patients contractant le COVID-19 dans le monde était de 5821739 dont 358104 (6,15%) décédés et 2522202 (43,32%) guéries [4]. Le taux de mortalité spécifique du COVID-19 est variable [5]. Alors que le taux global est d’environ 2,3%, il atteint 8,0% et 14,8% chez les patients âgés, respectivement, de 70 à 79 ans et ≥ 80 ans [5]. Le diagnostic positif du COVID-19 repose sur un ensemble d´éléments regroupant la notion de contact avec un cas suspect/confirmé de COVID-19, les résultats des prélèvements virologiques, et la présence de signes cliniques et radiologiques évocateurs [6-10]. Les examens virologiques consistent en un test d’acide nucléique SRAS-CoV-2 (par écouvillonnage naso-pharyngée ou d´autres échantillons des voies respiratoires supérieures) et/ou un test sérologique des immunoglobulines (IgM et IgG) qui a une spécificité > 95% pour le COVID-19 [8,11]. Une méta-analyse récente a rapporté la proportion des signes cliniques dus au COVID-19 [12]. Il apparait que les principaux signes sont la fièvre, la toux, la myalgie, l´asthénie, la dyspnée, les céphalées, les odynophagies et les signes gastro-intestinaux. Cependant, l´analyse des caractéristiques cliniques et démographiques des patients COVID-19 de par le monde, a permis d´observer une sémiologie plus riche, différente d´un pays à un autre [6,13-17]. Si certains patients présentaient un tableau clinique clair avec le concept de contact avec un cas suspect/confirmé de COVID-19, d’autres ont des manifestations cliniques suggestives, indépendamment des antécédents de contagion [18]. A titre d´exemple, une anosmie/agueusie sans obstruction nasale était rapportée d´une manière fréquente [19]. Les fréquences des anomalies biologiques des formes symptomatiques du COVID-19 sont aussi très variables d´une étude à une autre [6].

D´une part, les fréquences des élévations de la C-réactive protéine (CRP), des transaminases [alanine et aspartate amino-transférase (ALAT, ASAT, respectivement)] et de la lactico-deshydrogénase (LDH), étaient notées, respectivement, chez 61-86%, 25%, et 13-98% des patients [6]. D´autre part, l´anémie, la thrombopénie et l´insuffisance rénale aiguë apparaissaient peu fréquentes [6]. La tomodensitométrie (TDM) thoracique joue un rôle clé dans le diagnostic rapide de la pathologie respiratoire et permet ainsi une prise en charge précoce [20]. Les signes radiologiques les plus observées sont les opacités en verre dépoli et les condensations parenchymateuses [6,20]. Comme pour les signes cliniques et les anomalies biologiques, la fréquence des signes radiologiques suggestives de COVID-19 est variable d´une étude à une autre [6,21]. Selon l’OMS, près de 190000 personnes pourraient mourir du COVID-19 en Afrique si la pathologie n’est pas maîtrisée [22]. L’Algérie comme le reste du monde est confrontée à la propagation de cette pathologie, et le premier patient atteint de cette virose était détecté le 25 février 2020. A la date du 28 mai 2020, le nombre de patients Algériens atteints de COVID-19 était de 8857 dont 623 décédés (7,0%) et 5129 (57,9%) guéries [23]. Pour bien lutter contre cette épidémie, les autorités sanitaires Algériennes ont besoin d´identifier le profil clinique (par ex; âge, sexe, notion de contact avec un cas suspect/confirmé de COVID-19, principales plaintes cliniques) et biologique (par ex; présence ou non d´un syndrome inflammatoire biologique (SIB), d´une altération des fonctions rénales et/ou hépatiques) des patients hospitalisés pour Covid-19. De même, étant donné que la gravité du COVID-19 consiste en une altération de la fonction respiratoire [5,17,24], il est primordial d’analyser les caractéristiques radiologiques de ces patients. La rapidité et l´étendue de la propagation virale du SARS-CoV-2 à travers le monde a conduit à de nombreuses publications évaluant les données clinique, biologique et radiologique propres à chaque pays/régions [2,5,6,16,17,24-27]. Il s´en sort que le COVID-19 est une pathologie systémique avec des réponses différentes selon les pays. En effet, il semble exister différents phénotypes de patients atteints de COVID-19 [28]. Cependant, aux meilleures des connaissances des auteurs, aucune étude antérieure n´a élaboré le profil clinique, biologique et tomodensitométrique des patients Algériens hospitalisés pour COVID-19. Ainsi, l´objectif de la présente étude était de déterminer les données préliminaires des patients Algérois hospitalisés pour COVID-19 durant le pic de l´épidémie.

## Méthodes

**Type d´étude:** Il s´agissait d´une étude prospective monocentrique. Elle était réalisée à l’hôpital universitaire de Rouiba, Alger (Algérie) durant la période allant du 19 mars au 30 avril 2020. L´hôpital de Rouiba draine les patients habitants la région Est d´Alger. L´étude était réalisée conformément aux principes de la Déclaration d’Helsinki et les données des patients étaient dépersonnalisées. Cette cohorte prospective comporte deux parties. La première est le sujet de cette étude. L´objectif de la deuxième partie sera de comparer les données des patients ayant une saturation pulsée de l´hémoglobine en oxygène (SpO_2_) > 95 et ≤ 95%.

**Figure 1:**
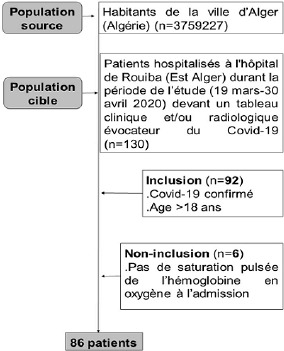
Organigramme de l’étude

**Population à l´étude, critères d´inclusion et de non-inclusion:** la Figure 1 expose l´organigramme de l’étude. La population source était les habitants de la région Est d´Alger (n=3759227). La population cible était les patients hospitalisés durant la période de l´étude devant un tableau clinique et tomodensitométrique évocateur du COVID-19. Seuls les patients ayant un diagnostic positif de COVID-19 confirmé par une sérologie (IgM, IgG) et/ou par une RT-PCR (Reverse transcription polymerase chain reaction) et une TDM thoracique compatible avec l´infection étaient inclus dans l’étude. L´absence de la mesure de la SpO_2_ à l´admission était appliquée comme un critère de non-inclusion.

**Test RT-PCR et tests sérologiques de l´infection COVID-19:** des écouvillons naso-pharyngés étaient prélevés à l’admission au niveau des services dédiés à la prise en charge des patients suspectés de COVID-19 (par ex: pneumologie et médecine interne de l’hôpital de Rouiba, Alger). La mise en évidence du matériel génomique du coronavirus était réalisée au niveau du laboratoire central de l’hôpital de Rouiba, par la PCR en temps réel (RT-PCR, méthode de référence du diagnostic moléculaire du SARS-CoV-2) [8]. Le test sérologique de l´infection COVID-19 permet une détection qualitative des IgG et/ou des IgM dans le sérum, le sang total ou le plasma humain en 10 à 15 minutes [7,8]. Ce test combiné IgG - IgM, a une sensibilité de 88,66% et une spécificité de 90,63% [8]. L’utilisation simultanée de la PCR et des tests d’anticorps améliore le diagnostic aux différents stades de la pathologie [7,8].

**Données cliniques:** la collecte des données était réalisée d´une manière prospective à partir d´une fiche d´observation préétablie et des dossiers des patients hospitalisés. Les données cliniques suivantes étaient collectées: âge (an), sexe, tabagisme actif, notion de contact avec un cas suspect ou confirmé de COVID-19, délai diagnostic (intervalle entre la date d´apparition des symptômes et la date d´hospitalisation), antécédents médicaux des patients [par ex, hypertension artérielle (HTA), diabète sucré (DS), cardiopathie, asthme, bronchopneumopathie chronique obstructive (BPCO), allergie], principales plaintes, durée de séjour à l’hôpital. Deux groupes d´âge étaient définis: ≥ 50 et < 50 ans [13,16]. La SpO_2_ à l´admission était déterminée au repos et à l´air ambiant (Bedside monitor, Nihon Kohden Corporation Model BSM-3562, Japon). En Algérie, le protocole thérapeutique recommandé par le ministère de la santé, de la population et de la réforme hospitalière [29] comporte l´association «hydroxychloroquine- azithromycine». Le traitement de base était l´association «Chloroquine - Azithromycine - Zinc - Vitamine C» et selon la sévérité de la pathologie deux types de traitements étaient identifiés: i) traitement de base et enoxaparine à titre préventif; et ii) traitement de base, enoxaparine à titre curatif, double antibiothérapie et ± une corticothérapie. Le nombre (%) des patients ayant nécessité une oxygénothérapie était noté.

**Données biologiques et définitions appliquées:** un prélèvement sanguin était réalisé afin de déterminer un bilan biologique standard comportant les données suivantes: numération formule sanguine (NFS) [hémoglobine (g/dl), leucocytes (10^3^/mm^3^), formule leucocytaire (10^3^/mm^3^) [polynucléaires neutrophiles, éosinophiles et basophiles (PNN, PNE, PNB, respectivement), lymphocytes et monocytes], plaquettes (10^3^/mm^3^)], CRP (mg/L), vitesse de sédimentation à la première heure (VS, mm), fonction rénale (urée (g/l), créatinine (mg/l)), fonction hépatique (transaminases (UI/L), phosphatase alcaline (PAL, UI/L)), ionogramme sanguin (kaliémie et natrémie, (mmol/l)), taux de prothrombine (TP), créatine phosphokinase (CPK) et LDH. Les analyses étaient réalisées selon les méthodes habituelles du service de biologie de l’hôpital Rouiba d´Alger. L´anémie et la polyglobulie étaient définies devant une hémoglobinémie, respectivement, < 12 chez les femmes et < 13 chez les hommes, et > 17 [30]. Trois groupes de patients étaient définis [anémique; non-anémique; polyglobulie]. L´hyperleucocytose et la leucopénie étaient définies devant un nombre de leucocytes, respectivement, > 11 et < 4 [31]. Trois groupes étaient définis [leucopénie; leucocytes dans les normes; hyperleucocytose]. La lymphopénie était retenue devant un nombre de lymphocytes < 1000 [31]. La basocytémie était retenue devant un nombre de PNB > 150 [31]. La thrombopénie et la thrombocytose étaient définies devant des nombres de plaquettes, respectivement, < 150 et > 450 [31]. Un TP < 70% était qualifié de diminué [32]. La CRP était considérée comme augmentée si le taux était > 12 mg/L [33]. Chez les patients âgés < 50 ans, des VS > 15 chez les hommes ou > 20 chez les femmes étaient considérées comme augmentées [34]. Chez les patients âgés ≥ 50 ans, des VS > 20 chez les hommes ou > 30 chez les femmes étaient considérées comme augmentées [34]. Le SIB était défini devant une CRP et/ou une VS augmentées et deux groupes étaient définis [pas de SIB; présence d´un SIB]. L´hyper-urémie était retenue devant une valeur > 0,45 [35,36], et l´hyper-créatininémie était retenue devant une valeur > 14 [35,36]. La cytolyse hépatique était retenue devant des valeurs d´ALAT et/ou d´ASAT > 50 [37]. La PAL était considérée comme élevée devant un taux > 460 UI/L [38]. L´hypo- et l´hyper- kaliémie étaient retenues devant des kaliémies, respectivement, < 3,3 et > 5,1 [39]. L´hypo- et l´hyper- natrémie étaient retenues devant des natrémies, respectivement, < 135 et > 145 [40]. Les taux de LDH [marqueur de dommage tissulaire (nécrose, hypoxie, hémolyse, infarctus du myocarde) [41] et de CPK (marqueur de myolyse [42]) étaient considérés comme élevés devant des valeurs supérieures, respectivement, à 460 UI/L [43] et 170 UI/L [44].

**Données tomodensitométriques du thorax:** une TDM thoracique, sans produit de contraste, était réalisée. Des évaluations visuelles semi-quantitatives des images tomodensitométriques étaient réalisées par un radiologue expérimenté (FS dans la liste des auteurs). Les signes radiologiques suivants étaient classés comme compatibles avec la pneumonie COVID-19: opacités en verre dépoli multifocales périphériques avec ou sans réticulation, condensation parenchymateuse, aspect en mosaïque [21,45]. La topographie et la localisation des signes radiologiques étaient précisées. L´extension des lésions radiologiques était évaluée dans chaque lobe selon une échelle allant de 0 à 5 [15]: 0 (pas de signe TDM), 1 (atteinte < 10%), 2 (atteinte de 10-25%), 3 (atteinte de 25-50%), 4 (atteinte de 50-75%) et 5 (atteinte > 75%). L´estimation de l´extension pulmonaire totale était harmonisée: légère (stades 0-2: <25%), modérée (stade 3), sévère (stade 4), et critique (stade 5) [46].

**Analyses statistiques:** l´analyse de la distribution des variables était réalisée grâce au test de Kolmogorov-Smirnov. La distribution des variables quantitatives était normale et les résultats étaient exprimés par leurs moyennes±écart-types. Les données qualitatives étaient exprimées en nombre (%). Les données manquantes étaient supprimées des analyses statistiques [14]. La saisie des résultats était réalisée en utilisant le logiciel Statistica (Statistica Kernel version 6; Stat Soft. France).

## Résultats

Sur les 130 patients hospitalisés dans le service, 92 étaient confirmés COVID-19. Parmi ces derniers, six étaient exclus car il manquait la SpO_2_ à l´admission. Les 86 patients retenus étaient âgés entre 21 et 84 ans (Figure 1).

**Profil clinique:** la moyenne±écart-type (minimum-maximum) de la SpO_2_ était de 95±3 (81-99) et 35 (40,7%) patients avaient une SpO_2_ ≤ 95%. Le profil clinique des patients atteints de COVID-19 était un homme non-fumeur, âgé de 53 ans, qui était dans 41,9% des cas en contact avec un cas suspect ou confirmé de COVID-19, et qui avait un délai diagnostic de 10 jours. Il s´agissait dans 70% des cas d´un patient suivi pour une HTA et/ou un DS et/ou une pathologie respiratoire chronique et/ou une allergie et/ou une cardiopathie. Les signes cliniques étaient dominés par la triade «asthénie-fièvre-toux» dans plus de 70% des cas (Tableau 1).

**Tableau 1:** Caractéristiques, antécédents et plaintes des patients (n=86)*

Caractéristiques	
Age (ans)	53±15 (20-84)
Age ≥ 50 ans	47 (54,6)
Sexe (femme)	34 (39,5)
Fumeur	15 (16,3)
Contact avec un cas suspect/confirmé de Covid-19	36 (41,9)
Délai diagnostic (jour)	10±7 (0-30)
Antécédents (par ordre de fréquence)	
Hypertension artérielle	27 (31,4)
Diabète sucré	13 (15,1)
Pathologie respiratoire chronique et allergie	11 (12,0)
Cardiopathie	9 (10,5)
Pathologie cancéreuse	5 (5,4)
Pathologie thyroïdienne	2 (2,2)
Pathologie psychiatrique	2 (2,2)
Pathologie neurologique	2 (2,2)
Autres (ostéoporose et insuffisance rénale chronique)	2 (2,2)
Plaintes (par ordre de fréquence)	
Asthénie	69 (80,2)
Fièvre	64 (74,4)
Toux	61 (70,9)
Céphalée	45 (52,3)
Anorexie	43 (50,0)
Myalgie	41 (47,7)
Agueusie	37 (43,0)
Anosmie	31 (37,0)
Diarrhée	33 (38,4)
Dyspnée	25 (29,1)
Odynophagie	16 (18,6)
Vomissements	7 (7,6)
Douleur thoracique	5 (5,8)
Hémoptysie	2 (2,2)
Douleur abdominale	2 (2,2)
Brulure oculaire	1 (1,1)
Rhinorrhée	1 (1,1)
Vertige	1 (1,1)

Les données quantitative et qualitative étaient exprimées en moyenne ± écart-type (minimum-maximum) et en nombre (%), respectivement.

* Le délai diagnostic manquait pour 23 patients.

**Profil biologique:** les anomalies biologiques les plus fréquentes étaient les suivantes: SIB (90,1%), VS augmentée (84,4%), basocytémie (70,8%), CRP augmentée (53,5%), lymphopénie (53,3%), dommage tissulaire (52,2%), anémie (38,7%), myolyse (28,8%), cytolyse hépatique (27,6%), hyponatrémie (20,3%), hyper-urémie (20,0%) et un TP diminué (19,4%) (Tableau 2 et Tableau 3).

**Tableau 2:** Données de la NFS, de la VS et de la CRP des patients (n=86)*

Hémoglobine (g/dl)	12,96±1,92
Leucocytes (103/mm3)	6962±2980
**Formule leucocytaire**	
PNN (103/mm3)	4661±2602
PNE (103/mm3)	22±30
PNB (103/mm3)	253±168
Lymphocytes (103/mm3)	982±523
Monocytes (103/mm3)	1006±585
Plaquettes (103/mm3)	276±106
VS (mm) (1ère h)	71±44
CRP (mg/L)	52±64
**Profil des patients (par ordre de fréquence)**	
Syndrome inflammatoire biologique	64 (90,1)
VS (1ère h) augmentée	54 (84,4)
Basocytémie	51 (70,8)
CRP augmentée	38 (53,5)
Lymphopénie	40 (53,3)
Anémie	29 (38,7)
Hyperleucocytose	9 (12,0)
Leucopénie	8 (10,7)
Thrombopénie	5 (6,8)
Thrombocytose	5 (6,8)
Polyglobulie	2 (2,7)

CRP: C-réactive protéine. NFS: numération formule sanguine. PNB: polynucléaire basophile. PNE: polynucléaire éosinophile. PNN: polynucléaire neutrophile. VS: vitesse de sédimentation. Les données quantitative et qualitative étaient exprimées en moyenne ± écart-type et en nombre (%), respectivement.

* Données manquantes [variable (nombre de patients)]: hémoglobine (11), leucocytes (11), lymphocytes (11), PNN (12), monocytes (12), plaquettes (12), PNB (14), PNE (14), CRP (15), syndrome inflammatoire (15), VS (22).

**Tableau 3:** Données biologiques des patients (n=86)*

Données biologiques		
Fonction rénale	Urée (g/L)	0,35±0,17
	Créatinine (mg/L)	10,38±3,00
Fonction hépatique	ASAT (UI/L)	50±36
	ALAT (UI/L)	45±46
	PAL (UI/L)	164±65
Ionogramme sanguin	Potassium (mmol/l)	3,92±0,51
	Sodium (mmol/l)	137,21±18,11
TP (%)		79±11
CPK (UI/L)		188±426
LDH (UI/L)		579±311
**Profil biologique des patients (par ordre de fréquence)**		
LDH augmentée (dommage tissulaire)		36 (52,2)
CPK augmentée (myolyse)		17 (28,8)
Cytolyse hépatique		21 (27,6)
Hyponatrémie		12 (20,3)
Hyper-urémie		15 (20,0)
TP diminué		12 (19,4)
Hyper-créatininémie		8 (10,7)
Hypokaliémie		3 (5,1)
Hypernatrémie		3 (5,1)
Hyperkaliémie		2 (3,4)
PAL augmentée		0 (0,0)

ALAT: alanine amino-transférase. ASAT: aspartate amino-transférase. CPK: créatine phosphokinase. LDH: lactico-deshydrogénase. PAL: phosphatse alacaline. TP: taux de prothrombine. Les données quantitative et qualitative étaient exprimées en moyenne±écart-type et en nombre (%), respectivement.

* Données manquantes [variable (nombre de patients)]: transaminases (10), fonction rénale (11), PAL (11), LDH (17), TP (24), CPK (27), ionogramme sanguin (27).

**Profil radiologique:** les quatre signes radiologiques les plus fréquents étaient les suivants: aspect en verre dépoli (91,8%), condensations alvéolaires (61,2%), aspect en verre dépoli en plage (60,0%), et aspect en verre dépoli nodulaire (55,3%). La topographie la plus fréquente était la région sous-pleurale (90,6), et les lésions étaient bilatérales dans 82,4% des cas. L´extension sévère était présente dans 11,8% des cas (Tableau 4)

**Tableau 4:** Données tomodensitométriques du thorax des patients (n=85)*

Signes radiologiques (par ordre de fréquence)	
Verre dépoli	78 (91,8)
Condensation alvéolaire	52 (61,2)
Verre dépoli en plage	51 (60,0)
Verre dépoli nodulaire	47 (55,3)
Aspect en mosaïque	34 (40,0)
Condensation en bande	34 (40,0)
Condensation nodulaire	29 (34,1)
Syndrome bronchique	5 (5,9)
Epanchement pleurale	1 (1,2)
Embolie pulmonaire	1 (1,2)
Emphysème	1 (1,2)
Nodule sous pleural	1 (1,2)
Topographie des lésions (par ordre de fréquence)	
Sous pleurale	77 (90,6)
Bilatérale	70 (82,4)
Prédominance inférieure	52 (61,2)
Mixte	34 (40,0)
Péri-bronchovasculaire	15 (17,6)
Extension des lésions	
Légère	53 (62,3)
Modérée	22 (25,9)
Sévère	10 (11,8)

Les données étaient exprimées en nombre (%).

* Seuls 85 patients ont réalisé un scanner thoracique.

**Traitements reçus et durée d´hospitalisation:** le traitement de base associé à l´enoxaparine préventive était prescrit chez 65,1% des patients. L´oxygénothérapie était prescrite chez 12,8% des patients et la moyenne de la durée d´hospitalisation était de 7 jours. Durant la période d´étude, un décè était noté [pathologie de Waldenstrom] (Tableau 5).

**Tableau 5:** Traitements reçus et durée d’hospitalisation des patients (n=86)

Chloroquine + Azithromycine + Zinc + Vitamine C	+ Enoxaparine préventive	56 (65,1)
	+ Enoxaparine curative + Double antibiothérapie ± Corticoïde	30 (34,9)
Oxygénothérapie		11 (12,8)
Durée d’hospitalisation (jour)		7±3 (1-15)

Les données quantitative et qualitative étaient exprimées en moyenne ± écart-type et en nombre (%), respectivement.

## Discussion

Aux meilleures des connaissances des auteurs, il s´agit de la première étude qui détermine le profil des patients Algériens hospitalisés pour COVID-19. Cette étude confirme encore une fois que le COVID-19 est une pathologie qui a plusieurs visages [47]. En effet, elle est une pathologie complexe, qui fait intervenir des phases virale, inflammatoire et thrombotique [47]. La compréhension des présentations cliniques, biologiques et radiologiques des infections à coronavirus est indispensable pour le diagnostic, l´appréciation de la gravité de la pathologie et aussi pour l´évaluation de la réponse au traitement et le suivi.

**Profil clinique:** dans la littérature, il existe une hétérogénéité des données démographiques dans les populations de patients COVID-19 [6,13,14,^16^,^17^]. La prédominance masculine observait dans cette étude est intermédiaire avec celles rapportaient dans la littérature [13,48]. D´une part, le sex-ratio (homme/femme) variait de 1,4 [14] à 1,8 [16], et d´autre part, 58 à 82% des patients étaient des hommes [14,16,17,48], et ce pourcentage n´était que de 45% dans la population des 20 patients Tunisiens [13]. Ces différences pourraient s’expliquer par la fréquence élevée des facteurs de risques de sévérité du COVID-19 dans la population masculine [6]. Seul 16,3% des patients Algériens étaient des fumeurs actifs. Ceci confirme les données de la littérature puisque les fumeurs représentaient 6 [14,17] à 35% [13] des patients hospitalisés. Dans une méta-analyse incluant 11590 patients, 6,3% étaient des fumeurs [49]. La même étude a montré que le tabagisme était un facteur de risque de progression du COVID-19 (comparativement aux non-fumeurs, les fumeurs avaient 1,91 fois plus de chances de progression de la gravité du COVID-19 [49]). Dans la présente étude, la moyenne d´âge était de 53 ans et 54,6% des patients étaient âgés ≥ 50 ans. D´une part, ces données sont intermédiaires avec celles rapportées dans quelques études [14,25-27,50] où la médiane d´âge variait de 44 [26] à 57 [25] ans. D´autre part, ces données sont différentes de celles Tunisiennes où l´âge variait de 41 à 85 ans et où 75% des patients étaient âgés > 50 ans [13]. Dans cette étude, 41,9% des patients avaient un contact avec un cas suspect/confirmé de COVID-19. Dans la littérature, différents pourcentages étaient rapportés: 38% [17], 44% [14], 49,3% [16], 58% [13]. Le délai diagnostic noté dans cette étude était proche de ceux rapportés dans la littérature [14,51,52]. En effet, la majorité des patients développaient des symptômes dans les 11,5 [52] et 12,5 [51] jours précédant l´hospitalisation.

Les comorbidités sont des facteurs de risque possibles d’augmentation de la sévérité du COVID-19. Dans l´étude Tunisienne [13], 80% des patients avaient une pathologie chronique, et l´HTA était la comorbidité la plus fréquente (55%). Dans les études similaires [13,14,16,17], les principales comorbidités retrouvées dans la population des patients hospitalisés étaient l´HTA (15-55%), le DS (7,4-30%) et les pathologies vasculaires (2,5-15%). La fréquence des pathologies cancéreuses notées dans cette étude est largement supérieure à celles rapportées dans la littérature (0,5 [16], 0,9 [14] et 1% [17]). Le COVID-19 se manifeste essentiellement par une atteinte respiratoire, mais une sémiologie plus riche commence à être rapportée [6]. Dans cette étude, les plaintes étaient dominées par la triade «asthénie-fièvre-toux». Dans les études similaires [13,14,16,17], les signes cardinaux du COVID-19 associaient une fièvre (88,7-100%), une toux (67,8-85%), des expectorations (23-41,3%) et une dyspnée (18,7-85%). Dans cette étude, alors que l´asthénie était le signe le plus fréquemment rapporté (80,2%), la dyspnée (29,1%) était classée en dixième position. La fréquence de l´asthénie rapportée dans cette étude est proche de celle observée dans l´étude Tunisienne (70%) [13]. La céphalée était le 4ème signe rapporté par les patients, avec une fréquence (52,3%) proche de celle observée dans l´étude Tunisienne (55%) [13]. Les signes digestifs notés dans cette étude et dans la littérature [par ex, anorexie (84%) [53], diarrhée (3,8-15%) [6,13], nausées/vomissements (4-5%) [6] et douleur abdominale (25%) [53]] peuvent inaugurer le tableau clinique [6]. La myalgie était le sixième signe rapporté par les patients Algériens, avec une fréquence (47,7%) nettement supérieure à celle rapportée dans la littérature (15-32%) [6].

Concordant avec les données de la littérature [6,13], 43,0 et 37,0% des patients Algériens présentaient, respectivement, une agueusie et une anosmie. Une augmentation des consultations médicales pour anosmie/agueusie sans obstruction était rapportée dans le contexte de cette pandémie [6]. Dans cette étude, et similaire à l´étude Tunisienne [13], 18,6 et 20% des patients présentaient une odynophagie. La douleur thoracique était rapportée par 5,8% des patients Algériens. Dans la littérature, une souffrance myocardique était retrouvée chez 10-20% des patients [6]. De même, il semble que 49% des patients décédés du COVID-19 présentaient une défaillance cardiaque, et la moitié étaient indemnes de pathologie cardiovasculaire [10]. Dans cette étude, des rares signes cliniques [par ex; hémoptysie, brulure oculaire, rhinorrhée et vertige] étaient rapportés. Dans le COVID-19, l´hémoptysie était décrite comme un symptôme peu fréquent (0-5%) [27,54]. Concernant les atteintes ophtalmologiques, des lésions à type de conjonctivite étaient décrites, et semblaient être associées aux formes sévères du COVID-19 [55]. Dans l´étude de Guan et al. [14], 4,8% des patients Chinois avaient une rhinite. Dans la littérature, certains patients présentaient un tableau neurologique [par ex; confusion (14,8%), atteinte neuromusculaire (19,3%), accidents vasculaires cérébraux (5,7%) [56]. Enfin, des lésions cutanées (par ex; maculopapuleuses érythémateuses ou à types d´engelures) et des réactions urticariennes étaient évoquées mais leurs associations au COVID-19 n´étaient pas confirmées [6].

**Profil biologique:** les fréquences des anomalies biologiques rapportées dans cette étude (Tableau 2 et Tableau 3) sont intermédiaires avec celles observées dans la littérature [6,13,14,16,17]. En effet, différentes fréquences étaient rapportées: SIB (en particulier une CRP augmentée, 35-85,6%) [13,16], lymphopénie (40-83,2%) [14,16,17], dommage tissulaire (41-98%) [14,16], anémie (0-15%) [13,17], myolyse (4,5-13,7%) [14,16], cytolyse hépatique (15-31%) [13,17], hyponatrémie (2,5-50%) [57], hyper-urémie (4,5%) [16], TP diminué (2,1-94%) [16,17]. Dans cette étude d´autres anomalies biologiques, moins fréquentes, étaient rapportées (par ex; hyperleucocytose, hyper-créatininémie, leucopénie, thrombocytose, thrombopénie, hypernatrémie, hypokaliémie, hyperkaliémie, et polyglobulie). Dans la littérature, différentes fréquences de ces anomalies étaient rapportées [6,13,14,16,17]: hyperleucocytose (5,9%) [14], hyper-créatininémie (1,6-10%) [13,14], leucopénie (33,7%) [14], thrombocytose (0) [13], thrombopénie (0-36,2%) [13,14], hypernatrémie (4-57%) [57,58], hypokaliémie (10-15%) [13], et hyperkaliémie (23%) [57]. L’hypernatrémie et l’hypokaliémie sont courantes dans le syndrome de détresse respiratoire aiguë sévère du COVID-19 [28,58].

Il est possible que la pathogenèse du virus et l’hyperaldostéronisme secondaire consécutive causée par une augmentation des niveaux des récepteurs d´angiotensine-II soient responsables du résultat [59]. La polyglobulie n´a pas été rapportée dans la littérature, mais une étude en cours avance l´hypothèse que les lésions du COVID-19 peuvent entraîner une polycythémie secondaire qui serait interprétée comme une pathologie chronique des montagnes [60]. Autrement dit, le COVID-19 est une pathologie qui simule une exposition extrême à haute altitude [60]. D´autres anomalies biologiques, non évaluées dans cette étude, étaient rapportées dans la littérature [6]. Il s´agit de l´hypoalbuminémie [16,17], de l´hyperferritinémie (78,5%-80%) [16,17], de l´hyperbilirubinémie (5,1-10,5%) [14,16], de l´hyperglycémie (45,2-52%) [16,50], de l´alcalose respiratoire (28%) [61], des élévations des D-dimères (23,3-46,4%) [14,16,17] ou des troponines (17%) [17]. L´hyperglycémie peut être expliquée par l´hypersécrétion de glucocorticoïdes endogènes secondaire dans le contexte de stress induit par l´infection ou par l´utilisation de corticoïdes à visée thérapeutique [6].

**Profil radiologique:** la TDM à une place prépondérante dans le diagnostic initial et l´évaluation de l´éxtension de l´atteinte respiratoire [20]. Etant donné le caractère non spécifique des signes radiologiques, la TDM thoracique est préconisée en cas de probabilité pré-test élevée (par ex; devant des signes cliniques évocateurs chez un patient hospitalisé présentant une forme sévère du COVID-19) [6]. Les fréquences des signes radiologiques notées dans cette étude (Tableau 4) sont intermédiaires avec celles observées dans la littérature [13-17]. En effet, dans les études similaires [13-15], les fréquences des signes radiologiques étaient très variables: verre dépoli (56,4-97,6%) [13-15], opacités linéaires (65,1%) [15], foyer de condensation (33,3-63,9%) [13,15], épaississements des septa inter-lobulaires (62,7%) [15], condensations alvéolaires bilatérales (51,8-55,5%) [13,14], condensations alvéolaires unilatérales (41,9%) [14], aspect en mosaïque (36,1%) [15], signe de la toile d’araignée (opacité en verre dépoli sous pleurale, entourée de réticulations interlobulaires [6], 25,3%) [15], épaississements des parois bronchiques (22,9%) [15], embolie pulmonaire (22,2%) [13], épaississements sous-pleuraux (20,5%) [15], anomalies interstitielles (14,7%) [15], adénopathie médiastinale (8,4%) [15], épanchements pleuraux (8,4%) [15], épanchements péricardiques (4,8%) [15]. Les complications thromboemboliques sont dues à un état d´hypercoagulabilité accompagnant surtout les formes modérées et graves [62]. Dans la présente étude, la topographie la plus fréquente était la localisation sous-pleurale et les lésions étaient souvent bilatérales (Tableau 4). Dans les études similaires, les localisations les plus fréquentes étaient le lobe moyen (73,5%) [15] et le lobe supérieur droit (64,7%) [15] et les atteintes étaient bilatérales dans 95,2% des cas [15]. Dans cette étude, l´extension pulmonaire légère dominait le tableau radiologique (Tableau 4). Aux meilleures des connaissances des auteurs, aucune étude antérieure n´a étudié l´extension des lésions pulmonaires. La divergence dans les tableaux radiologiques est expliquée, en partie, par le délai de réalisation de la TDM par rapport aux premiers symptômes [9]. En effet, au stade initial, il y a une prédominance d´images en verre dépoli, qui évoluent vers une association de verre dépoli, d´opacité réticulaires et de foyers de condensation à un stade avancé [9].

**Traitements reçus et durée d´hospitalisation:** l´association «chloroquine-azithromycine» était prescrite chez tous les patients, et seuls 34,9% recevaient une double antibiothérapie plus ou moins des corticoïdes (Tableau 5). L´association «chloroquine-azithromycine» est un protocole thérapeutique adopté par de nombreux pays [63,64]. A titre d´exemple, 75% des patients Tunisiens recevaient cette association [13]. De même, dans la présente même étude [13], avant leur admission, 35% des patients recevaient une antibiothérapie à large spectre. Dans un souci de standardisation de la prise en charge thérapeutique, le schéma thérapeutique était adapté à la sévérité de l´atteinte. L´évaluation des résultats est en cours. L´oxygénothérapie était prescrite chez 12,8% des patients Algériens. Cette fréquence est nettement inférieure à celle rapportée dans la littérature (90 [13], 48,7 [16], 41,3 [14], 21% [17]). La moyenne de la durée d´hospitalisation de cette étude (7 jours) est plus basse que celle rapportée dans la littérature (9 [65], 10 [66], 14 [24] jours).

**Limites méthodologiques:** cette étude présente quelques limites méthodologiques. La première concerne la durée limitée de l´étude (42 jours) d´où l´impossibilité d´apercevoir l´évolution de la pathologie à moyen terme. Cette limite était imposée par le contexte épidémique de la pandémie actuelle. La deuxième limite concerne les données manquantes qui étaient acquises dans des conditions de crise [14,16,17]. Par conséquent, l’exhaustivité du recueil des données, en particulier au moment de l’admission à l’hôpital, n’était pas optimale. Cependant, plusieurs tentatives pour compléter au maximum le recueil des données (par ex, sollicitations des équipes soignantes prenant en charge les patients, appels téléphoniques des contacts ou l´entourage) étaient essayées. La troisième limite concerne l´application de différents seuils pour classer certains paramètres biologiques comme anormaux. Malgré la consultation étoffée de la littérature, ils n´existaient pas des standards relatifs aux normalités des valeurs de certains paramètres biologiques étudiés permettant la comparaison des résultats de manière uniformisée. A titre d´exemple, alors que dans cette étude un taux de LDH (UI/l) > 460 était considéré augmenté et un taux de plaquettes (10^3^/mm^3^) < 150 retenait la thrombopénie, dans d´autres études des seuils différents étaient appliqués pour la LDH (> 250 [14] ou > 100 [16]) et pour retenir la thrombopénie (< 180 [16] ou < 100 [17]). Ces situations rendent difficile les comparaisons entre les études. La quatrième limite concerne le caractère monocentrique de l´étude. D´une part, ceci pourrait entraver les perspectives de généralisation des résultats à l´ensemble des patients Algériens hospitalisés pour COVID-19. D´autre part, les données étaient recueillies chez des patients hospitalisés et décrivaient donc des formes modérées à graves de la pathologie [6]. Selon la littérature, il semble que 80% des patients présentaient des symptômes bénins et donc ne nécessitaient pas une hospitalisation [5]. De ce fait, il est capital de mentionner que les caractéristiques des patients hospitalisés sont différentes de celles nécessitant une prise en charge en soins intensifs [6]. A titre d´exemple, sur les 1591 patients Italiens hospitalisés en réanimation, 82% étaient des hommes, 49% avaient une HTA, 21% étaient suivis pour une pathologie cardiovasculaire, 17% pour un DS, 8% pour une néoplasie, 4% pour une BPCO, et 3% pour une insuffisance rénale chronique [48]. Cette limite était imposée par le contexte de l´étude en situation de crise avec la nécessité de sa réalisation dans des situations d’urgence.

## Conclusion

La cible principale du virus SARS-CoV-2 est le poumon, mais une atteinte multi systémique est possible. Cette étude a déterminé, dans le contexte pandémique actuel, le profil clinique, biologique et radiologique des patients Algériens hospitalisés pour COVID-19.

**Perspectives:** il serait souhaitable de réaliser une étude Algérienne à plus large échelle et multicentriques. Ceci confèrera plus d´arguments justifiant la généralisation des résultats à l´échelle nationale et contribuera ainsi à l´émission, par les autorités sanitaires Algériennes, de recommandations standards. Ces dernières contribueront à une meilleure prise en charge et une prévention de ce nouveau virus émergeant. De même, comme une basse SpO_2_ au moment du diagnostic suggère une forme sévère du COVID-19 [24], il serait intéressant de comparer les profils des patients répartis selon un seuil de SpO_2_ (par ex; < 95% [24]).

### Etat des connaissances actuelle sur le sujet

- Le 12 mars 2020, l’OMS a déclaré le COVID-19 comme une pandémie;
- L’analyse des caractéristiques clinique, biologique et radiologique des patients COVID-19 de par le monde, a permis d’observer une sémiologie riche, différente d’un pays à un autre;
- Aux meilleures des connaissances des auteurs, aucune étude antérieure n’a déterminé le profil clinique, biologique et tomodensitométrique des patients Algériens hospitalisés pour COVID-19.

### Contribution de notre étude à la connaissance

- Le profil clinique des patients Algériens atteints de COVID-19 était un homme non-fumeur, âgé de 53 ans, qui était dans 42% des cas en contact avec un cas suspect/confirmé de COVID-19, qui avait un délai diagnostic de 10 jours, ayant au moins une comorbidité dans 70% des cas et qui présentait la triade «asthénie-fièvre-toux» dans plus de 70% des cas;
- Les anomalies biologiques les plus fréquentes (>50%) étaient: syndrome inflammatoire biologique (90,1%), vitesse de sédimentation augmentée (84,4%), basocytémie (70,8%), C-réactive protéine augmentée (53,5%), lymphopénie (53,3%), dommage tissulaire (52,2%);
- Les signes radiologiques les plus fréquents (>50%) étaient: verre dépoli (91,8%), condensations alvéolaires (61,2%), verre dépoli en plage (60,0%) et verre dépoli nodulaire (55,3%).

## Conflits d’intérêts

Les auteurs ne déclarent aucun conflit d´intérêts.

## Remerciements

Les auteurs dédient cet article aux personnes qui ont consacré leurs vies pour lutter contre le coronavirus, et à tous les professionnels de la santé qui ont pris soins des patients COVID-19. Les auteurs remercient aussi les responsables des centres d´imagerie médicale (Cimagerie) à Rouiba et de diagnostic médical (Kacel) à Reghaia, pour leurs contributions à titre gracieux dans la prise en charge de patient COVID-19.
